# Risk of intraocular hemorrhage among exudative age-related macular degeneration patients treated with different anti-VEGF agents

**DOI:** 10.1371/journal.pone.0356596

**Published:** 2026-08-27

**Authors:** Min Seok Kim, Seonghee Nam, Jeongwoo Lee, Se Joon Woo

**Affiliations:** 1 Department of Ophthalmology, Seoul National University College of Medicine, Seoul National University Bundang Hospital, Seongnam, Korea; 2 Data research, Samil Pharm Co. LTD, Seoul, Korea; Akita University: Akita Daigaku, JAPAN

## Abstract

**Background:**

Intraocular hemorrhage is a vision-threatening complication in patients with exudative age-related macular degeneration (AMD) receiving intravitreal anti-vascular endothelial growth factor (VEGF) therapy. We aimed to compare the risk of intraocular hemorrhage requiring vitrectomy among patients with exudative AMD treated with ranibizumab, aflibercept, or brolucizumab.

**Methods and findings:**

This nationwide retrospective cohort study used claims data from the Korean National Health Insurance Review and Assessment Service (May 2014–April 2023). Korean patients aged 40 years or older with newly diagnosed exudative AMD who initiated anti-VEGF therapy and had no history of intraocular hemorrhage were included. The primary outcome was intraocular hemorrhage requiring vitrectomy after anti-VEGF initiation. Time-to-event analyses using Cox proportional hazards regression estimated hazard ratios (HRs) and 95% CIs; Kaplan–Meier curves showed cumulative incidence probability, and logistic regression provided complementary risk estimates.

Among 75,812 patients, 1,128 (1.5%) experienced intraocular hemorrhage requiring vitrectomy. Compared with ranibizumab, the adjusted risk was higher with brolucizumab (HR, 1.83; 95% CI, 1.18–2.83; *P* = .007) and aflibercept (HR, 1.31; 95% CI, 1.14–1.49; *P* < .001). Logistic regression similarly showed higher odds with aflibercept versus ranibizumab (adjusted odds ratio, 1.32; 95% CI, 1.16–1.51; *P* < .001). Patients with hemorrhage received fewer anti-VEGF injections in the first year than those without hemorrhage (3.15 ± 1.87 vs 4.01 ± 1.85; *P* < .001).

**Conclusion:**

In this large nationwide cohort, intraocular hemorrhage requiring vitrectomy after anti-VEGF therapy for exudative AMD was rare but occurred more frequently with brolucizumab and aflibercept than with ranibizumab. Although unlikely to be the primary determinant of anti-VEGF selection, these findings provide adjunctive safety information that may be considered when counseling and monitoring selected patients.

## Introduction

Age-related macular degeneration (AMD) is a leading cause of irreversible visual impairment in the elderly population worldwide. The neovascular or exudative form of AMD is characterized by abnormal choroidal neovascularization, which can lead to subretinal or intraretinal fluid accumulation, hemorrhage, and eventually fibrosis and central vision loss.[1] The advent of anti–vascular endothelial growth factor (VEGF) therapy has revolutionized the treatment of exudative AMD, significantly improving visual outcomes and reducing the rate of severe vision loss.[2–4]

Among the anti-VEGF agents currently used, ranibizumab, aflibercept, and brolucizumab are the most widely approved and prescribed.[3,5,6] While these agents are generally effective and well tolerated, concerns remain about rare but serious adverse events, including intraocular hemorrhage.[7,8]

Despite the clinical importance of such complications, real-world comparative data on the incidence of hemorrhagic events across different anti-VEGF agents remain limited. The Health Insurance Review and Assessment (HIRA) database of Korea, which includes comprehensive nationwide health claims data, provides a unique opportunity to investigate these events in a large cohort.

In this study, we aimed to compare the risk of vitrectomy due to vitreous or retinal hemorrhage following anti-VEGF therapy among patients with exudative AMD treated with ranibizumab, aflibercept, or brolucizumab in a real-world setting using the HIRA database.

## Methods

### Ethics statement

This study was approved by the Institutional Review Board of Seoul National University Bundang Hospital (approval no. X-2312-868-902). The study complied with the tenets of the Declaration of Helsinki and followed good clinical practice guidelines. The requirement for informed consent was waived because this study used deidentified claims data.

### Data source

We conducted a retrospective cohort study using data from the HIRA service of Korea (project no. M20231203002). HIRA is a national claims database covering approximately 97% of the Korean population, and it includes detailed information on diagnoses, procedures, prescriptions, demographics, and healthcare utilization. The data used in this study were accessed for research purposes on November 4, 2025.

### Study population

Patients with exudative AMD were identified using the special registration code for exudative AMD (V201) between May 1, 2014, and April 30, 2023. To ensure a newly diagnosed cohort, we excluded individuals diagnosed with exudative AMD during a one-year washout period from May 1, 2014, to April 30, 2015. Patients younger than 40 years were also excluded.

Additional exclusion criteria included: (1) never receiving any anti-VEGF injections during the study period, (2) receiving anti-VEGF treatment prior to the initial diagnosis of exudative AMD, and (3) experiencing the outcome event prior to the first anti-VEGF injection. The cohort was divided into three groups based on the anti-VEGF agent first initiated: ranibizumab, aflibercept, or brolucizumab.

### Outcome definition

The primary outcome was defined as undergoing a vitrectomy procedure (surgical codes S5121 for total vitrectomy or S5122 for partial vitrectomy) in conjunction with a diagnostic code for vitreous hemorrhage (H431, H450) or retinal hemorrhage (H356) after the initiation of anti-VEGF therapy.

### Statistical analysis

Time-to-event analysis was conducted using Cox proportional hazards regression to estimate hazard ratio (HR) for the occurrence of intraocular hemorrhage requiring vitrectomy among the three treatment groups. Three models were constructed: Model 1, Crude, unadjusted comparison; Model 2, Adjusted for sex and age; Model 3, Adjusted for sex, age, comorbidities, and the use of antithrombotic agents. Comorbidities included hypertension, diabetes mellitus, dyslipidemia, heart failure, atrial fibrillation, myocardial infarction, ischemic stroke, peripheral artery disease, and cancer. The antithrombotic agents considered in the analysis included warfarin, sulfomucopolysaccharide, sulodexide, novel oral anticoagulants, aspirin, P2Y12 inhibitors, selexipag, beraprost sodium, mesoglycan sodium, triflusal, sarpogrelate, cilostazol, and dipyridamole. We considered the use of antithrombotic agents as a time-varying covariate starting from the first anti-VEGF injection.

Patients were followed from the date of first anti-VEGF injection until the date of the outcome event or end of the study period, whichever came first. Patients who switched to another anti-VEGF agent during follow-up were also censored at the time of switching. During follow-up, 14,061 patients (18.5%) switched anti-VEGF agents; among them, 8,509 (60.5%) were initially treated with ranibizumab, 5,432 (38.6%) with aflibercept, and 120 (0.9%) with brolucizumab.

Kaplan–Meier survival curves were plotted to visualize the cumulative incidence probability of intraocular hemorrhage requiring vitrectomy for each treatment group, and differences were assessed using the log-rank test. Brolucizumab was approved in Korea in 2021, resulting in a relatively short follow-up duration. Therefore, the comparison among the three agents was limited to outcomes within one year after the first injection. In contrast, the comparison between ranibizumab and aflibercept utilized data from the entire study period.

Additionally, we performed logistic regression analyses using the occurrence of intraocular hemorrhage requiring vitrectomy as the dependent variable, applying the same three models used in the Cox regression analysis.

All statistical analyses were performed using SAS version 9.4 (SAS Institute, Cary, NC) and R version 4.0.3 (The R Foundation for Statistical Computing, Vienna, Austria). A two-sided p-value < 0.05 was considered statistically significant.

## Results

**Fig 1:**
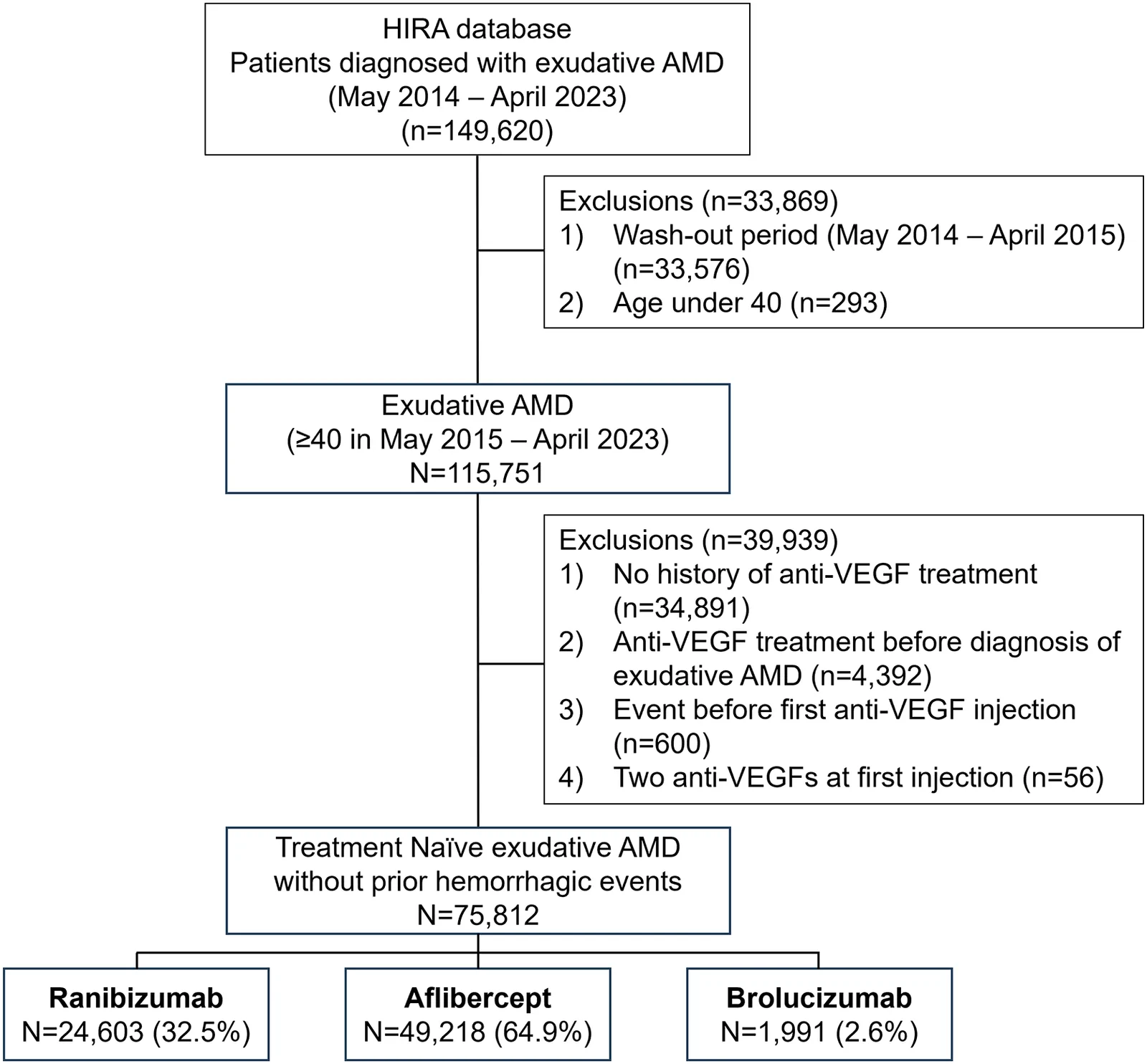
Flow diagram.

A total of 149,620 patients were diagnosed with exudative AMD between May 2014 and April 2023. After applying exclusion criteria, a total of 75,812 treatment-naïve patients aged 40 years or older were included in the cohort (Fig 1).

These patients were categorized into three treatment groups based on their initial anti-VEGF agent: ranibizumab (n = 24,603; 32.5%), aflibercept (n = 49,218; 64.9%), and brolucizumab (n = 1,991; 2.6%). Table 1 summarizes the baseline characteristics of the study population.

**Table 1:** Baseline and clinical characteristics.

	Total (N = 75,812)	Ranibizumab (N = 24,603)	Aflibercept (N = 49,218)	Brolucizumab (N = 1,991)	P
Sex					
Men	46396 (61.2)	13665 (55.5)	31385 (63.8)	1346 (67.6)	<.001
Women	29416 (38.8)	10938 (44.5)	17833 (36.2)	645 (32.4)	
Age	71.5 ± 9.4	72.5 ± 9.4	71.0 ± 9.4	71.1 ± 9.1	<.001
Age group					
40-49	665 (0.9)	211 (0.9)	436 (0.9)	18 (0.9)	<.001
50-59	8137 (10.7)	2260 (9.2)	5685 (11.6)	192 (9.6)	
60-69	21449 (28.3)	6166 (25.1)	14654 (29.8)	629 (31.6)	
70-79	29366 (38.7)	9976 (40.5)	18625 (37.8)	765 (38.4)	
≥80	16195 (21.4)	5990 (24.3)	9818 (19.9)	387 (19.4)	
Follow up duration (year)	2.8 ± 2.2	2.8 ± 2.3	2.9 ± 2.1	0.8 ± 0.5	<.001
Comorbidities					
Hypertension	48519 (64)	16270 (66.1)	30972 (62.9)	1277 (64.1)	<.001
Diabetes mellitus	18217 (24.0)	6046 (24.6)	11674 (23.7)	497 (25.0)	0.023
Dyslipidemia	36133 (47.7)	11792 (47.9)	23254 (47.2)	1087 (54.6)	<.001
Heart failure	4857 (6.4)	1597 (6.5)	3117 (6.3)	143 (7.2)	0.255
Atrial fibrillation	2830 (3.7)	941 (3.8)	1794 (3.6)	95 (4.8)	0.022
Myocardial infarction	1076 (1.4)	368 (1.5)	681 (1.4)	27 (1.4)	0.465
Ischemic stroke	5332 (7.0)	1883 (7.7)	3315 (6.7)	134 (6.7)	<.001
Peripheral artery disease	16375 (21.6)	5477 (22.3)	10446 (21.2)	452 (22.7)	0.003
Cancer	7635 (10.1)	2461 (10.0)	4943 (10.0)	231 (11.6)	0.07
Exposure to antithrombotic drugs	34829 (45.9)	11986 (48.7)	22220 (45.2)	623 (31.3)	<.001
Intraocular hemorrhage requiring vitrectomy	1128 (1.5)	300 (1.2)	806 (1.6)	22 (1.1)	<.001

Among all patients, 1,128 individuals (1.5%) experienced intraocular hemorrhage requiring vitrectomy: 300 patients (1.2%) in the ranibizumab group, 806 patients (1.6%) in the aflibercept group, and 22 patients (1.1%) in the brolucizumab group. The mean number of anti-VEGF injections before the event was 4.3 ± 4.3 over a mean duration of 541 ± 610 days. Specifically, it was 4.2 ± 3.9 injections over 607 ± 634 days in the ranibizumab group, 4.4 ± 4.5 injections over 530 ± 602 days in the aflibercept group, and 1.5 ± 0.9 injections over 59 ± 46 days in the brolucizumab group. The number of injections was significantly lower in the intraocular hemorrhage group than in the non-hemorrhage group across all follow-up years (year 1: 3.15 ± 1.87 vs. 4.01 ± 1.85; *P* < 0.001) (S1 Table).

Cox proportional hazards regression analysis demonstrated that, compared to ranibizumab, the risk of intraocular hemorrhage requiring vitrectomy was higher in patients treated with brolucizumab (adjusted HR, 1.83; 95% CI, 1.18–2.83; *P* = 0.007) and aflibercept (adjusted HR, 1.31; 95% CI, 1.14–1.49; *P* < 0.001), indicating a graded increase in risk across the three agents (Table 2).

**Table 2:** Cox proportional hazards regression for intraocular hemorrhage requiring vitrectomy.

	Model 1		Model 2		Model 3	
	HR (95% CI)	*P*	HR (95% CI)	*P*	HR (95% CI)	*P*
Anti-VEGF						
Ranibizumab	reference		reference		reference	
Aflibercept	1.33 (1.17-1.52)	<.001	1.30 (1.14-1.48)	<.001	1.31 (1.14-1.49)	<.001
Brolucizumab	1.87 (1.21-2.89)	0.005	1.82 (1.18-2.82)	0.007	1.83 (1.18-2.83)	0.007
Sex						
Men	–	–	reference		reference	
Women	–	–	0.95 (0.84-1.07)	0.373	0.95 (0.84-1.08)	0.459
Age group						
40-49	–	–	reference		reference	
50-59	–	–	1.84 (0.86-3.92)	0.114	1.76 (0.83-3.76)	0.143
60-69	–	–	1.68 (0.79-3.54)	0.176	1.51 (0.72-3.20)	0.279
70-79	–	–	1.46 (0.69-3.08)	0.323	1.26 (0.60-2.67)	0.545
≥80	–	–	1.25 (0.59-2.67)	0.558	1.08 (0.50-2.30)	0.852
Comorbidities						
Hypertension	–	–	–	–	1.08 (0.93-1.25)	0.332
Diabetes mellitus	–	–	–	–	1.21 (1.05-1.39)	0.007
Dyslipidemia	–	–	–	–	1.09 (0.95-1.25)	0.212
Heart failure	–	–	–	–	0.96 (0.74-1.24)	0.734
Atrial fibrillation	–	–	–	–	1.24 (0.92-1.68)	0.158
Myocardial infarction	–	–	–	–	1.27 (0.82-1.97)	0.286
Ischemic stroke	–	–	–	–	0.94 (0.75-1.19)	0.622
Peripheral artery disease	–	–	–	–	0.99 (0.85-1.15)	0.897
Cancer	–	–	–	–	0.80 (0.64-1.00)	0.049
Exposure to antithrombotic agents	–	–	–	–	1.20 (1.04-1.39)	0.014

Diabetes mellitus (adjusted HR, 1.21; 95% CI, 1.05–1.39; *P* = 0.007) and exposure to antithrombotic agents (adjusted HR, 1.20; 95% CI, 1.04–1.39; *P* = 0.014) were also identified as risk factors for intraocular hemorrhage requiring vitrectomy, whereas cancer showed a borderline inverse association (adjusted HR, 0.80; 95% CI, 0.64–1.00; *P* = 0.049).

**Fig 2:**
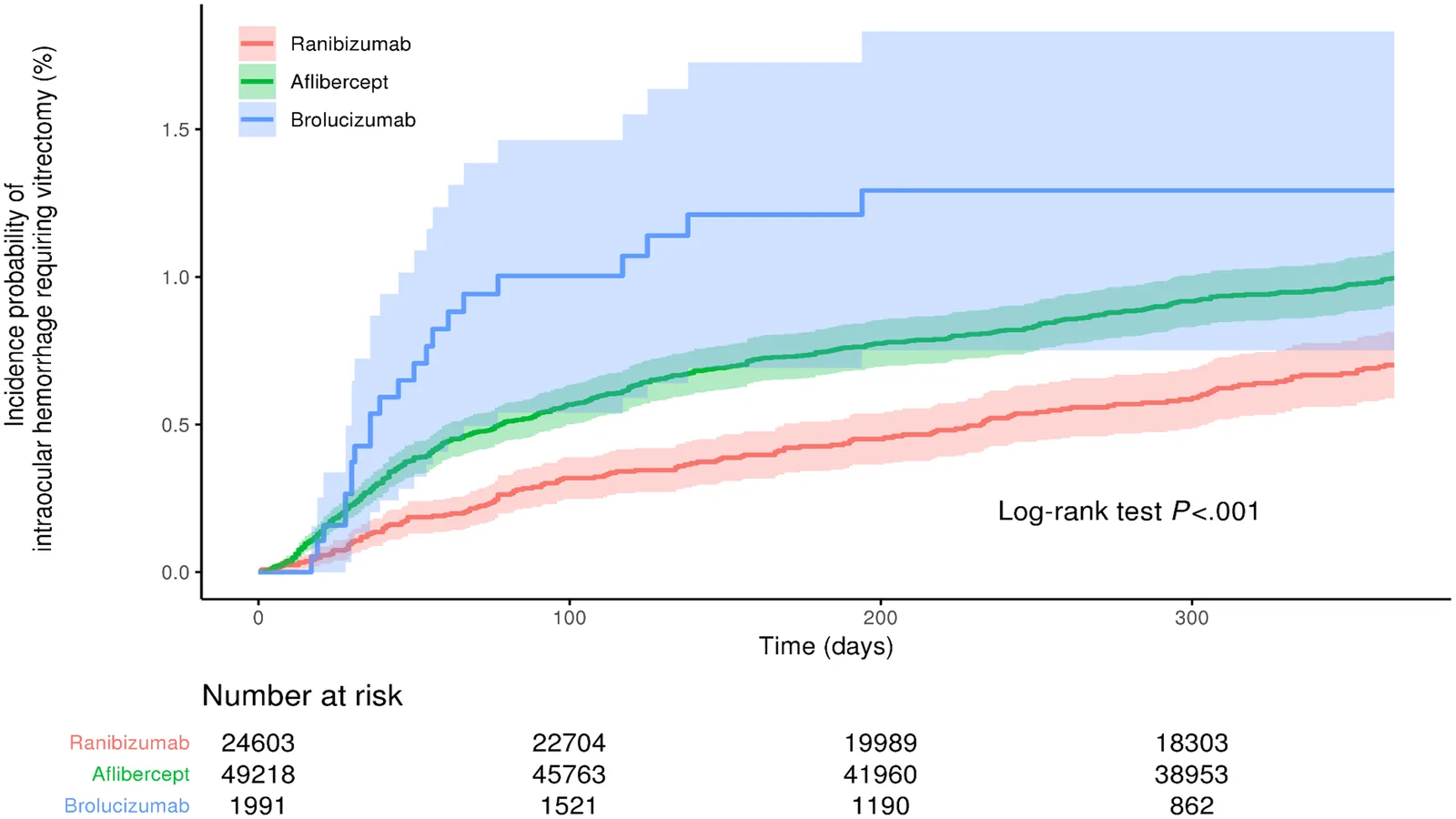
Kaplan-Meier curve comparing ranibizumab, aflibercept, and brolucizumab for 1 year.

In the Kaplan–Meier survival curves, the cumulative incidence probability of intraocular hemorrhage requiring vitrectomy was highest in the brolucizumab group, followed by aflibercept and ranibizumab during the one-year follow-up (Fig 2).

**Fig 3:**
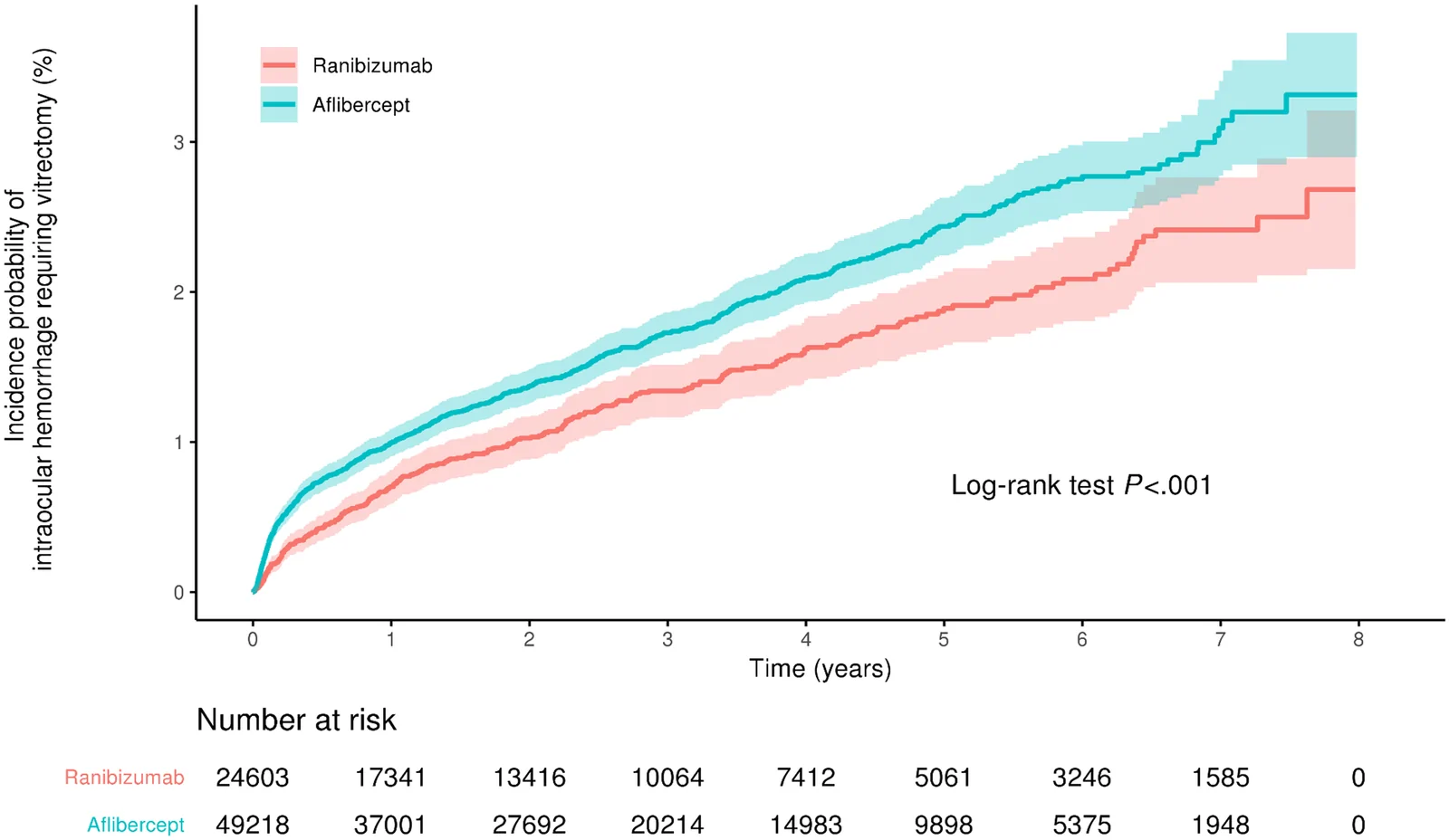
Kaplan-Meier curve comparing ranibizumab and aflibercept for 8 years.

In the comparison between aflibercept and ranibizumab, aflibercept was associated with higher risk of the intraocular hemorrhage requiring vitrectomy (Fig 3).

The log-rank test confirmed statistically significant differences between groups (*P* < 0.001). In logistic regression analysis, aflibercept was associated with a significantly higher risk of intraocular hemorrhage requiring vitrectomy compared to ranibizumab (adjusted odds ratio [OR], 1.32; 95% CI, 1.16–1.51; *P* < 0.001), whereas brolucizumab was not (adjusted OR, 0.89; 95% CI, 0.57–1.37; *P* = 0.582) (Table 3).

**Table 3:** Logistic regression analysis for intraocular hemorrhage requiring vitrectomy.

	Model 1		Model 2		Model 3	
	OR (95% CI)	*P*	OR (95% CI)	*P*	OR (95% CI)	*P*
Anti-VEGF						
Ranibizumab	reference		reference		reference	
Aflibercept	1.35 (1.18-1.54)	<.001	1.32 (1.15-1.50)	<.001	1.32 (1.16-1.51)	<.001
Brolucizumab	0.91 (0.59-1.40)	0.656	0.88 (0.57-1.37)	0.578	0.89 (0.57-1.37)	0.582
Sex						
Men	–	–	reference		reference	
Women	–	–	0.97 (0.86-1.10)	0.605	0.97 (0.86-1.10)	0.664
Age group						
40-49	–	–	reference		reference	
50-59	–	–	1.83 (0.85-3.92)	0.12	1.77 (0.83-3.80)	0.141
60-69	–	–	1.58 (0.75-3.35)	0.233	1.47 (0.69-3.13)	0.316
70-79	–	–	1.39 (0.66-2.94)	0.392	1.25 (0.59-2.66)	0.558
≥80	–	–	1.10 (0.51-2.34)	0.816	0.98 (0.45-2.09)	0.947
Comorbidities						
Hypertension	–	–	–	–	1.15 (1.00-1.33)	0.058
Diabetes mellitus	–	–	–	–	1.23 (1.07-1.41)	0.004
Dyslipidemia	–	–	–	–	1.01 (0.88-1.15)	0.913
Heart failure	–	–	–	–	0.93 (0.72-1.21)	0.592
Atrial fibrillation	–	–	–	–	1.25 (0.92-1.69)	0.151
Myocardial infarction	–	–	–	–	1.30 (0.83-2.02)	0.251
Ischemic stroke	–	–	–	–	1.02 (0.81-1.30)	0.843
Peripheral artery disease	–	–	–	–	0.99 (0.86-1.15)	0.924
Cancer	–	–	–	–	0.75 (0.60-0.94)	0.012

## Discussion

In this study, we compared the risk of vitreous or retinal hemorrhage requiring vitrectomy among patients with exudative AMD treated with ranibizumab, aflibercept, or brolucizumab using the HIRA database. Our findings demonstrate that, compared to ranibizumab, both aflibercept and brolucizumab were associated with higher risk of intraocular hemorrhage requiring vitrectomy, with a graded increase in hazard ratio across the three agents. Notably, brolucizumab showed the highest risk, followed by aflibercept and ranibizumab.

Breakthrough vitreous hemorrhage secondary to submacular hemorrhage in exudative AMD has been consistently reported in previous studies, with generally poor visual prognosis.[9] In particular, the incidence of vitreous hemorrhage in the polypoidal choroidal vasculopathy (PCV) subtype has been reported to range from 12.4% to 19.9%.[10,11]

In a retrospective cohort study using a representative national U.S. database, 140,915 eyes with neovascular AMD were analyzed, of which 9,107 eyes (6.46%) were diagnosed with submacular hemorrhage following the initiation of anti-VEGF therapy.[12] Among these cases, 158 eyes (1.7%) underwent vitrectomy within 30 days of the hemorrhage. However, due to a high rate of treatment switching, the authors assessed the incidence of submacular hemorrhage based on the last administered injection, which may not have adequately captured the true differences in hemorrhagic risk associated with each anti-VEGF agent.

Shin et al. reported that PCV subtype, large-diameter submacular hemorrhage, and anticoagulant use were significant risk factors for breakthrough vitreous hemorrhage following anti-VEGF injection in AMD patients with submacular hemorrhage, whereas the type of anti-VEGF agent was not.[8] However, the small sample size—only 31 cases and 87 controls—limits the clinical significance of their findings. Vitreous hemorrhage secondary to intravitreal injections were also reported in 0.1% among 44,734 injections in a single institution.[13]

Although it remains unclear whether intraocular hemorrhage occurs as part of the natural course of exudative AMD or whether anti-VEGF injections increase the risk of such hemorrhage, our study demonstrated that there is a difference in risk among the three anti-VEGF agents. All three agents neutralize VEGF, but they vary in molecular design and pharmacokinetics, which can affect their intravitreal behavior and potentially the vascular response. Ranibizumab is a humanized Fab fragment derived from monoclonal antibody targeting all VEGF-A isoforms, with a molecular weight of 48 kDa.[14,15] Aflibercept is a recombinant fusion protein (~115 kDa) composed of the key binding domains of VEGF receptors 1 and 2 fused to the Fc portion of human IgG. Aflibercept has a 94-fold higher binding affinity for VEGF-A compared to ranibizumab, and the VIEW 1 and VIEW 2 studies demonstrated non-inferior visual outcomes with fewer injections of aflibercept compared to ranibizumab.[3,16] Inflammatory or immunogenic reactions are rare with both ranibizumab and aflibercept.[3] Brolucizumab is a humanized single-chain variable fragment antibody that inhibits all VEGF-A isoforms. It is the smallest of the three agents (~26 kDa), allowing for a highest molar dose (12 times than aflibercept and 22 times than ranibizumab) to be delivered in the same injection volume.[17,18] Its high molar concentration and strong binding affinity were suggested to result in prolonged VEGF suppression. In clinical trials, approximately half of patients could be maintained on a 12-week dosing interval after the loading phase.[6] However, a notable drawback of brolucizumab is its higher rate of intraocular inflammation (IOI); in phase III trials, IOI occurred in 4.6% of brolucizumab-treated eyes, compared to 1.1% in the aflibercept group.[19] These pharmacologic differences can influence how each drug controls neovascular lesions and potentially how hemorrhagic complications develop. A more potent or longer-acting agent might better prevent recurrent exudation if dosing is optimized, but could also induce rapid vascular regression (e.g., collapse of a pigment epithelial detachment) that in rare cases precipitates a retinal pigment epithelium (RPE) tear and hemorrhage.[20] In the HAWK and HARRIER trials, RPE tears occurred in 2.5% of eyes treated with brolucizumab 6 mg, compared to 1.1% of those treated with aflibercept 2 mg.[6] Each drug’s immunogenic profile could also be relevant. Brolucizumab’s propensity for inflammatory reactions could indirectly affect the chorioretinal circulation and lead to vascular occlusion or secondary neovascular complications including subretinal hemorrhage.[21]

Beyond molecular properties, different hemorrhage risk among the anti-VEGF agents may be explained by how and in whom these drugs are used. Brolucizumab, due to its longer-lasting effect, is typically administered less frequently than ranibizumab or aflibercept. The frequency of injections could influence the risk of hemorrhagic complications, with less frequent injections possibly leading to more pronounced fluctuations in VEGF levels and vascular instability, especially in patients with the more advanced cases or different type of exudative AMD. For example, patients on aflibercept or brolucizumab may have a different form of AMD such as PCV that predisposes them to a higher hemorrhagic risk, which could amplify the differences in event risk. This limitation is particularly relevant in Asian populations, where PCV has been reported to account for approximately 22–62% of neovascular AMD cases. [22] Therefore, the inability to distinguish PCV from other subtypes represents an important limitation in the present study. Nevertheless, the nationwide design and large sample size reduce the likelihood that the observed associations are explained solely by differences in PCV distribution between treatment groups. Similarly, patients treated with ranibizumab may have had less severe status or different clinical characteristics at baseline, which could partially explain the lower observed hemorrhage rate.

There are several limitations to this study. First, the diagnostic codes used in the claims database do not differentiate subtypes of exudative AMD, particularly PCV, making subtype-specific analyses unfeasible. This is an important limitation because PCV was reported to have a higher risk of intraocular hemorrhage than typical neovascular AMD and may also influence treatment selection in routine clinical practice. Consequently, differences in the distribution of PCV across treatment groups could have introduced residual confounding and affected the observed hemorrhagic risk. Future studies incorporating imaging findings will be needed to determine whether the observed associations differ according to AMD subtype. Second, the brolucizumab group was substantially smaller than the ranibizumab and aflibercept groups and had a relatively short follow-up duration because of its later introduction into clinical practice. Therefore, comparisons among the three anti-VEGF agents were limited, and the findings for brolucizumab should be interpreted with caution. Further studies with larger sample sizes and longer follow-up are needed to confirm the long-term safety of brolucizumab. Third, the choice of the initial anti-VEGF agent may have been influenced by the severity or subtype of AMD in routine clinical practice, resulting in potential selection bias. Because these factors were not fully captured in the claims database, residual confounding could not be completely excluded despite adjustment for available covariates. Despite these limitations, this study is notable for being the first to compare the risk of hemorrhagic complications among the three commonly used anti-VEGF agents in patients with exudative AMD. Further studies involving diverse ethnic populations and longer follow-up periods in brolucizumab-treated patients are needed to validate our findings and draw definitive conclusions.

## Conclusions

In this large-scale Korean real-world cohort study, brolucizumab and aflibercept were associated with a higher risk of serious intraocular hemorrhage than ranibizumab in patients with exudative AMD. Although intraocular hemorrhage requiring vitrectomy is a rare event and unlikely to be the primary determinant of anti-VEGF selection in routine practice, our findings suggest that different anti-VEGF agents may confer varying risks of hemorrhagic complications. These results should be interpreted as complementary safety information to be weighed alongside efficacy, overall safety profile, treatment burden, and cost, and may help inform risk–benefit discussions and monitoring strategies in selected patients.

## Supporting information

S1 Table Number of anti-vascular endothelial growth factor injections during the follow up. (PDF)
