# Supplementary material for: Risk of intraocular hemorrhage among exudative age-related macular degeneration patients treated with different anti-VEGF agents
Source: PLoS One. 2026 Aug 27;21(8):e0356596. doi: 10.1371/journal.pone.0356596 (PMC13521369; doi:10.1371/journal.pone.0356596)
Supplement: Supplementary file 1 [file pone.0356596.s001.pdf]

S1 Table. Number of anti-vascular endothelial growth factor injections during the follow up

| | Total (N=75,812) | Intraocular hemorrhage (N=1,128) | Non-intraocular hemorrhage (N=74,684) | *P** |
|---|---|---|---|---|
| Year 1 | 4.00 ± 1.85 | 3.15 ± 1.87 | 4.01 ± 1.85 | <.001 |
| Year 2 | 5.00 ± 3.15 | 3.70 ± 2.83 | 5.02 ± 3.15 | <.001 |
| Year 3 | 5.63 ± 4.14 | 3.99 ± 3.48 | 5.66 ± 4.14 | <.001 |
| Year 4 | 6.05 ± 4.90 | 4.16 ± 3.90 | 6.08 ± 4.91 | <.001 |
| Year 5 | 6.34 ± 5.50 | 4.25 ± 4.14 | 6.37 ± 5.52 | <.001 |
| Year 6 | 6.49 ± 5.89 | 4.29 ± 4.28 | 6.53 ± 5.90 | <.001 |
| Year 7 | 6.57 ± 6.08 | 4.29 ± 4.28 | 6.60 ± 6.10 | <.001 |
| Year 8 | 6.59 ± 6.14 | 4.29 ± 4.28 | 6.62 ± 6.16 | <.001 |

*Mann-Whitney U test
